# Supplementary material for: Complexome profiling of the Chlamydomonas psb28 mutant reveals TEF5 as an early PSII assembly factor
Source: Plant Cell. 2025 Mar 17;37(6):koaf055. doi: 10.1093/plcell/koaf055 (PMC12164590; doi:10.1093/plcell/koaf055)
Supplement: koaf055_Supplementary_Data [file koaf055_supplementary_data.zip › psb28_interactive.zip!/plots/intensity/qualityPlots/peptides/HSP70A_Cre08.g372100.t1.2.html]

### Cre08.g372100.t1.2

Trivial name: HSP70A  
SeqCoverage [median]: 25.50  
Mass: 71.21 kDa  
  
 

| Sequence | MissedCleavage | Mass [Da] | wtIntensity | muIntensity | Score |
| --- | --- | --- | --- | --- | --- |
| AEEISSMVLIK | 0 | 1218.65 | 0.00 | 630.94 | 85.68 |
| AGAHDVPEIVVSYKNEEK | 1 | 1983.99 | 0.00 | 1833.10 | 73.50 |
| ATAGDTHLGGEDFDER | 0 | 1689.72 | 3814.13 | 7918.37 | 102.65 |
| AVVTVPAYFNDSQR | 0 | 1565.78 | 0.00 | 3328.56 | 94.30 |
| DAGMIAGLEVLR | 0 | 1243.66 | 0.00 | 3816.87 | 95.57 |
| DSGLGER | 0 | 732.34 | 0.00 | 1473.64 | 120.49 |
| HTVFDAK | 0 | 816.41 | 0.00 | 1919.28 | 92.47 |
| ITITNDKGR | 1 | 1016.56 | 13148.33 | 0.00 | 138.10 |
| KAVVTVPAYFNDSQR | 1 | 1693.88 | 0.00 | 510.27 | 48.41 |
| LVNHFANEFQR | 0 | 1373.68 | 773.04 | 3521.27 | 126.19 |
| NSLENYAYNMR | 0 | 1373.60 | 0.00 | 1840.64 | 138.11 |
| TTPSYVAFTDTER | 0 | 1486.69 | 8274.34 | 3766.89 | 223.14 |
|  |  |  | 26009.8 | 30559.8 |  |
